# Supplementary material for: Development of Single Nucleotide Polymorphism and Association Analysis with Growth Traits for Black Porgy (Acanthopagrus schlegelii)
Source: Genes (Basel). 2022 Oct 31;13(11):1992. doi: 10.3390/genes13111992 (PMC9690740; doi:10.3390/genes13111992)
Supplement: Supplementary file 1 [file genes-13-01992-s001.zip › genes-1908002-supplementary.pdf]

## Supplementary Tables

**Table S1.** Primer information of 61 candidate SNPs of black porgy.

| SNP | Mutated Base | Primer |
|---|---|---|
| scaffold2-2815335 | T→G | F：ACGTTGGATGTGAACCCCAGTGTGATTAGG<br>R：ACGTTGGATGTGAACCCCAGTGTGATTAGG<br>Extension：ACAGCTGAGAGAGCCCT |
| scaffold2-3426830 | T→C | F：ACGTTGGATGCACTCACACTGCTTGTAAAC<br>R：ACGTTGGATGCCTGCAGTTGTCAAAACCAC<br>Extension：ACACGGCTTGTAAACATCGTTGAC |
| scaffold2-13687576 | A→T | F：ACGTTGGATGATGCTGTTGCCATTACAGCC<br>R：ACGTTGGATGAAGTTGTCCCCAGGTTGATG<br>Extension：CAGCCTATTGCCTTTCAGT |
| scaffold3-16652593 | T→C | F：ACGTTGGATGAACTGTTAAAGGCACAGCCG<br>R：ACGTTGGATGATACCATCACCGATGGACAG<br>Extension：GGATAAGATCCTGGGAGCTGACAC |
| scaffold3-16653046 | C→T | F：ACGTTGGATGATTGTAGATGTGCCTGTCGG<br>R：ACGTTGGATGTGTACCTTTCCATCAATAGC<br>Extension：CGAAGAGCTTCTGGGCCGCGT |
| scaffold4-10430350 | A→G | F：ACGTTGGATGGGTAAAAGGTAAACATGCTCC<br>R：ACGTTGGATGACACCATTGTTGAGGCAAGG<br>Extension：AACTTTTGTTATTTTTCAGACAAATG |
| scaffold5-12042944 | T→C | F：ACGTTGGATGGGACCTCTTAAACTGACACG<br>R：ACGTTGGATGACTGCACACAGGACAGATAC<br>Extension：TCCACGCTTTGCGCTCCGC |
| scaffold6-5210078 | A→C | F：ACGTTGGATGTGCTTGATTTCGGACATGCC<br>R：ACGTTGGATGAGAGCCCCATGATGTACCC<br>Extension：GGGAGCAACTGGCGGGACAGGGC |
| scaffold6-9258715 | T→C | F：ACGTTGGATGAGGTGATGATGTGCAGACAG<br>R：ACGTTGGATGCAACTCCATGTTCGACATCC<br>Extension：CCGAAGCTGTCTCTTGTACTC |
| scaffold6-15802194 | C→T | F：ACGTTGGATGCACCATGAATGGAGAAGCTG<br>R：ACGTTGGATGTCTCCTCTTGTTGTTCAGGG<br>Extension：TTATCCCACACACCGGCCTC |
| scaffold7-15455173 | A→G | F：ACGTTGGATGTGTTTCAGACGTGCAAGCTC<br>R：ACGTTGGATGAAAAGCCTCCGGAGCAAATG<br>Extension：TGCCTCTTCTCTGCTCTCATCTT |
| scaffold7-15455275 | C→T | F：ACGTTGGATGGAAACAAGCTCTGAGTCTGG<br>R：ACGTTGGATGTTGATCTTGTCAGCTGGCAC<br>Extension：GAAGTGGAAGCCATCATGACTGA |
| scaffold7-20336421 | T→C | F：ACGTTGGATGTACCTATAGTTGGACACCGC<br>R：ACGTTGGATGTCCCGTGCTTCAGCTATGAG<br>Extension：TTGACGCACTGTCCTTT |
| scaffold8-9389475 | T→C | F：ACGTTGGATGTTTTTCAGCACGTCGCGAAG<br>R：ACGTTGGATGTCAGAGGTCTCCCTTCATTG<br>Extension：AGGCTCGAAGCCTCTTCTTCTC |
| scaffold8-12482471 | T→C | F：ACGTTGGATGACTTACTTGGACAGGCCGTG<br>R：ACGTTGGATGTTCTCCCAGATTCTGGAGAG<br>Extension：AGATGTCCTTGGTCATGGTGCC |

| | | |
|---|---|---|
| scaffold9-8466307 | T→C | F：ACGTTGGATGCCTCGTAAGCAGGATTCCAG |
| | | R：ACGTTGGATGTCTGTAGATGGACCAGTAGC |
| | | Extension：TGAGTCCAGCGCCCTCTATCAATGC |
| scaffold9-8467739 | G→A | F：ACGTTGGATGATTGAGTTCCAGGGATCCTC |
| | | R：ACGTTGGATGACAGGAAGCTTCGCAGTATG |
| | | Extension：TCCTGTTACTAAACCACAGTCAATCAT |
| scaffold10-3011393 | T→C | F：ACGTTGGATGAGACCAAACAGCTGACGGAG |
| | | R：ACGTTGGATGGAACTGTTCCGCCAGTTTGC |
| | | Extension：GGACCAGCTGACGGAGATGCTTCC |
| scaffold11-8650275 | A→G | F：ACGTTGGATGTTGATGCGACTGACAGAGAC |
| | | R：ACGTTGGATGGTTCTCGTCTTTGGTCTCAG |
| | | Extension：AAATGCGGGCCTTCGCGGA |
| scaffold12-12226863 | A→G | F：ACGTTGGATGTTCTTCTGCTGCTGGTCCTC |
| | | R：ACGTTGGATGAGTGTCCTGCTGCACCCTGA |
| | | Extension：AGGGTGCGGTGCTGCTGCCGGCG |
| scaffold12-12716321 | T→C | F：ACGTTGGATGTCGTGGAGCTCATAGAGAAG |
| | | R：ACGTTGGATGTCTCCGCTTGTTTGACTGTG |
| | | Extension：AGAAGAACTTGGACACGTC |
| scaffold12-12772854 | A→G | F：ACGTTGGATGTTGGCATCAACCTGGACAAC |
| | | R：ACGTTGGATGACTCCTTCTCAAAGGCATGG |
| | | Extension：CAGCCCTATAAGATGCC |
| scaffold13-4787950 | A→C | F：ACGTTGGATGTACCCTTTGGAACCACAAGC |
| | | R：ACGTTGGATGTTTGTACCCCATCCTTCCAC |
| | | Extension：CACAAGCCCATTTATCTC |
| scaffold13-4805623 | G→A | F：ACGTTGGATGGTTGCTTTCTGCTTTGCTCC |
| | | R：ACGTTGGATGGAAGTCTGTGACAGACTTGG |
| | | Extension：CTGCTTTGCTCCAGCTTT |
| scaffold13-5790209 | T→C | F：ACGTTGGATGACTCGTGAACGATGAGTGAC |
| | | R：ACGTTGGATGGCTACATGCTGGACAACTAC |
| | | Extension：AGGGACCCCCAGGTGGCAATCTG |
| scaffold13-6616349 | C→T | F：ACGTTGGATGTCATCCTGAGACCGGAGAAG |
| | | R：ACGTTGGATGTGTCGACTAGTCTGAGGAAG |
| | | Extension：GGGGTCATCTGGATCAAACTGGA |
| scaffold16-5142266 | T→C | F：ACGTTGGATGAGAACCATTGCAGCCAGATG |
| | | R：ACGTTGGATGGAAGTCTTGCTGGTTTCTCC |
| | | Extension：AGGTGAGCCCCAGCAGAGTG |
| scaffold16-5142293 | A→G | F：ACGTTGGATGTAGGCCTCACCTTTGATCTG |
| | | R：ACGTTGGATGTACCTGTGTGGTCACTCTGC |
| | | Extension：GGTTCAGTGGAGAGAACCAT |
| scaffold17-796332 | T→C | F：ACGTTGGATGCCTAGAGCCAGGATCTTATC |
| | | R：ACGTTGGATGCCCTGGAGTTCCACAAAGAA |
| | | Extension：CCTCATAACCATCAAAAATAAGGTC |
| scaffold17-796939 | T→C | F：ACGTTGGATGACAGAGCGTAGTGTATAACC |
| | | R：ACGTTGGATGCTATGACAACACAACAGTGC |
| | | Extension：CCCCTGTGTGTCAGCTTCCT |
| scaffold18-2467783 | T→C | F：ACGTTGGATGACACTCCTGCCTGTGTGGTC |
| | | R：ACGTTGGATGACTTGCACACACTCCATGAG |
| | | Extension：CCTGTCCGGAAACCAGGC |
| scaffold19-709400 | T→C | F：ACGTTGGATGACAATATCACTGGGTGGCTG |
| | | R：ACGTTGGATGGGCCAACAGCTTGTTTGAAG |
| | | Extension：TGGTGGCTGGACAAGAACAA |
| scaffold20-453978 | G→A | F：ACGTTGGATGGTCCCTCAATACAGGCTATG |

| | | |
|---|---|---|
| | | R：ACGTTGGATGCACATGTCGATACTTGTCTG |
| | | Extension：TATTTGATCGTTTGCTGACCATC |
| scaffold21-4955509 | A→G | F：ACGTTGGATGAACGTCAGGACGAGCCTGTG |
| | | R：ACGTTGGATGATCCCCTCAGAGTCTTTTGC |
| | | Extension：GGTGCAGGACGAGCCTGTGAGAGTGG |
| scaffold25-4206172 | A→G | F：ACGTTGGATGGTGTCCAGAACCTTCTGACC |
| | | R：ACGTTGGATGCTTCAGGTGAAAACACGGTC |
| | | Extension：ACACCAGACCCTCTGTGCC |
| scaffold31-9275712 | T→C | F：ACGTTGGATGCTTCCCCCGCATCACTTTAC |
| | | R：ACGTTGGATGTCAGAGAGATGAGGTGTGTG |
| | | Extension：GGTCACGCATCACTTTACCTCAGAA |
| scaffold31-11182212 | A→G | F：ACGTTGGATGGTGCTTGTTGCTCACCTTTG |
| | | R：ACGTTGGATGCCCAAGCTCAGACCTTGAAA |
| | | Extension：GCTCACCTTTGTCTTTCTT |
| scaffold31-12525471 | A→C | F：ACGTTGGATGTGTTGACAGCGTCTGCAATG |
| | | R：ACGTTGGATGGGCCAATCGTAACATTCAGC |
| | | Extension：TCCCGGTACTCGTCGGGATTTCT |
| scaffold34-3226435 | A→C | F：ACGTTGGATGCTGACGACATGGAGATGAAG |
| | | R：ACGTTGGATGGTGTTGACATTTACCTCTCC |
| | | Extension：CCCTGAGTATTCTGTGCACCCG |
| scaffold35-2164468 | A→G | F：ACGTTGGATGAAACTCACCTGCAGTTTGGC |
| | | R：ACGTTGGATGGTTGATTCCTGTTTGTGTGC |
| | | Extension：GATATCAGCCAGCTCTTT |
| scaffold36-4436178 | T→C | F：ACGTTGGATGCTGAGGACCATACAGCCATC |
| | | R：ACGTTGGATGCTCTGTTCTGTCCTTAGTCG |
| | | Extension：GCCTTGCAGCACATGGTACTCCGC |
| scaffold49-4261152 | G→A | F：ACGTTGGATGTCTCACCACACATGACGTTG |
| | | R：ACGTTGGATGACCATATTCATGGGTCACGG |
| | | Extension：ATGACGTTGTATAATTCTATATTTTC |
| scaffold59-960994 | A→G | F：ACGTTGGATGAGCTGGTGGATCGACCTGTG |
| | | R：ACGTTGGATGGGTTTAAACCTACGCCAGAG |
| | | Extension：AGCTTGTGGATCGACCTGTGGGACCC |
| scaffold65-275780 | A→G | F：ACGTTGGATGTCAGAGCCCTGATGAGGAAG |
| | | R：ACGTTGGATGGTGTCTTACTTCAGCTCCTG |
| | | Extension：AACAGGACAGAGTTGAGCAGAT |
| scaffold66-172323 | A→G | F：ACGTTGGATGGAGGCTGGTTTCTTTGTACG |
| | | R：ACGTTGGATGTGTTCATCACCCTCTCTCAC |
| | | Extension：GGTCAGGTATACAATCCAGTC |
| scaffold69-513313 | C→T | F：ACGTTGGATGACTGTCGAGTGCTACATGTC |
| | | R：ACGTTGGATGGAGAAAGAGAGAGATGCAGG |
| | | Extension：TTCATCCTCTTCATGCT |
| scaffold85-1906881 | A→G | F：ACGTTGGATGTGTATTTGCAGAGACCAGCG |
| | | R：ACGTTGGATGCTGAGGACCACATGAAGAAG |
| | | Extension：TGCGTAGAAAGGCATGTC |
| scaffold91-1266067 | A→G | F：ACGTTGGATGAGTCTGACACTCTGCAACTG |
| | | R：ACGTTGGATGCTTTGCTTTCCTCAGGTTTC |
| | | Extension：GATCCATGTTCTCTCACCAAG |
| scaffold102-1219145 | T→C | F：ACGTTGGATGAATCCTGCGCCTGAGAAGAC |
| | | R：ACGTTGGATGTTCTGCTCAGATTTGGGACG |
| | | Extension：CCATCCCGCTCGGGCATCGCTGCCTCC |
| scaffold102-1223941 | T→C | F：ACGTTGGATGCCAGATTAAAGCTGTGGTCC |
| | | R：ACGTTGGATGAAAGTGGCTGTGTAGCGTTG |

| | | |
|---|---|---|
| | | Extension：AAGCTGTGGTCCATAACTA |
| scaffold111-391534 | T→C | F：ACGTTGGATGAAGGAAAAACTGCAGCCGAG |
| | | R：ACGTTGGATGCTTACTTTGTGAGGCAGCAG |
| | | Extension：TCGTAAAGAGCTGGAGGC |
| scaffold141-357706 | T→C | F：ACGTTGGATGTGATGCGTGACTGCAGTTTC |
| | | R：ACGTTGGATGCAGGCCTGGCATCAAGATCA |
| | | Extension：CTGCTCTTGTTCTACAGGAGG |
| scaffold155-255306 | T→C | F：ACGTTGGATGTGTAACTTCAGGTGGGTCAG |
| | | R：ACGTTGGATGTGGAGAAGATGGGTTTGCAC |
| | | Extension：ATATCGAGGTGCGGCCTGGTAT |
| scaffold164-155638 | G→T | F：ACGTTGGATGACAAGCTGCTAGTAGGCAAC |
| | | R：ACGTTGGATGATGTCGTCTTACCTTCGCTG |
| | | Extension：CTCACAACGAAGAAGGT |
| scaffold168-178120 | A→G | F：ACGTTGGATGTGACTAACAGAAGACCGACC |
| | | R：ACGTTGGATGAAGCGTTTCTTCACCGTCAC |
| | | Extension：AACAACCGGCTCGCTTCTCCCC |
| scaffold184-113778 | C→T | F：ACGTTGGATGAAGAAGAACCCAGAGCAGTC |
| | | R：ACGTTGGATGGTCTGTTTGTCTCACCTTCC |
| | | Extension：CCCATCAGTCGGTCAACTTCGC |
| scaffold184-115262 | A→G | F：ACGTTGGATGCCTTTAGGTGGGATGTAGTC |
| | | R：ACGTTGGATGCAGCTGGTGATAAGAAGTGC |
| | | Extension：GGGATGTAGTCGCTCAT |
| scaffold210-68511 | A→G | F：ACGTTGGATGTTGTCCTGCAGACATTGCTC |
| | | R：ACGTTGGATGTCGACAACAGTAACGGTGAG |
| | | Extension：GGCCGGAAGTTTCATCAGCTGCACCA |
| scaffold290-11890 | T→C | F：ACGTTGGATGCCTCTGCTGAACTATTCACC |
| | | R：ACGTTGGATGGTACATCTGGGAGTCTCTTC |
| | | Extension：ATGTGTGATTCAGGTAAGGA |
| scaffold305-32581 | A→G | F：ACGTTGGATGACACCTTCTACAACGAGCTG |
| | | R：ACGTTGGATGTCTTCTCCCTGTTGGCCTTG |
| | | Extension：TACCACGAGCTGCGCGTGGCCCC |
| scaffold435-12868 | A→G | F：ACGTTGGATGCCAAATCTCAAGACCGTAGC |
| | | R：ACGTTGGATGACACATCCTTTGGGCTCATC |
| | | Extension：CCCGGACTGGGAAAGACAT |

**Table S2.** Genetic polymorphisms of black porgy SNP markers in the population.

| SNP | Genotypes | Genotype Frequency | Allele Frequency | *Ne* | *He* | *PIC* | $\chi^2$ |
|---|---|---|---|---|---|---|---|
| scaffold10-3011393 | CC | 0.867 | C: 0.927 | 1.156 | 0.135 | 0.131 | 1.879 |
| | CT | 0.120 | T: 0.073 | | | | |
| | TT | 0.013 | | | | | |
| scaffold10-3011393 | CC | 0.392 | C: 0.592 | 1.935 | 0.483 | 0.533 | 4.824 |
| | CT | 0.399 | T: 0.408 | | | | |
| | TT | 0.209 | | | | | |
| scaffold102-1223941 | CC | 0.943 | C: 0.946 | 1.113 | 0.102 | 0.100 | 138.966 |
| | CT | 0.006 | T: 0.054 | | | | |
| | TT | 0.051 | | | | | |
| scaffold11-8650275 | AA | 0.032 | A: 0.177 | 1.412 | 0.292 | 0.926 | 0.0004 |
| | AG | 0.291 | G: 0.823 | | | | |
| | GG | 0.677 | | | | | |
| scaffold111-391534 | CC | 0.759 | C: 0.858 | 1.323 | 0.244 | 0.235 | 6.114 |
| | CT | 0.196 | T: 0.142 | | | | |

| | | | | | | | |
|---|---|---|---|---|---|---|---|
| | TT | 0.044 | | | | | |
| scaffold12-12226863 | AG | 0.070 | A: 0.035 | 1.072 | 0.067 | 0.066 | 0.206 |
| | GG | 0.930 | G: 0.965 | | | | |
| | CC | 0.639 | C: 0.801 | 1.469 | 0.319 | 0.308 | 0.019 |
| scaffold12-12716321 | CT | 0.323 | T: 0.199 | | | | |
| | TT | 0.038 | | | | | |
| | AA | 0.095 | A: 0.256 | 1.616 | 0.381 | 0.862 | 3.484 |
| scaffold12-12772854 | AG | 0.323 | G: 0.744 | | | | |
| | GG | 0.582 | | | | | |
| | AA | 0.184 | A: 0.389 | 1.906 | 0.475 | 0.735 | 2.869 |
| scaffold13-4787950 | AC | 0.411 | C: 0.611 | | | | |
| | CC | 0.405 | | | | | |
| | AA | 0.070 | A: 0.522 | 1.996 | 0.499 | 0.603 | 104.610 |
| scaffold13-4805623 | AG | 0.905 | G: 0.478 | | | | |
| | GG | 0.025 | | | | | |
| | CC | 0.867 | C: 0.873 | 1.284 | 0.221 | 0.213 | 140.428 |
| scaffold13-5790209 | CT | 0.013 | T: 0.127 | | | | |
| | TT | 0.120 | | | | | |
| | CC | 0.044 | C: 0.108 | 1.238 | 0.192 | 0.970 | 18.355 |
| scaffold13-6616349 | CT | 0.127 | T: 0.892 | | | | |
| | TT | 0.829 | | | | | |
| | CC | 0.671 | C: 0.829 | 1.395 | 0.283 | 0.272 | 2.154 |
| scaffold141-357706 | CT | 0.316 | T: 0.171 | | | | |
| | TT | 0.013 | | | | | |
| | CC | 0.367 | C: 0.585 | 1.943 | 0.485 | 0.539 | 1.590 |
| scaffold155-255306 | CT | 0.437 | T: 0.415 | | | | |
| | TT | 0.196 | | | | | |
| | CC | 0.772 | C: 0.877 | 1.276 | 0.216 | 0.208 | 0.190 |
| scaffold16-5142266 | CT | 0.209 | T: 0.123 | | | | |
| | TT | 0.019 | | | | | |
| | AA | 0.114 | A: 0.320 | 1.770 | 0.435 | 0.803 | 0.463 |
| scaffold16-5142293 | AG | 0.411 | G: 0.680 | | | | |
| | GG | 0.475 | | | | | |
| | GG | 0.101 | G: 0.244 | 1.584 | 0.369 | 0.873 | 8.163 |
| scaffold164-155638 | TG | 0.285 | T: 0.756 | | | | |
| | TT | 0.614 | | | | | |
| | AA | 0.177 | A: 0.418 | 1.947 | 0.486 | 0.707 | 0.020 |
| scaffold168-178120 | AG | 0.481 | G: 0.582 | | | | |
| | GG | 0.342 | | | | | |
| | CC | 0.677 | C: 0.829 | 1.395 | 0.283 | 0.272 | 0.821 |
| scaffold17-796332 | CT | 0.304 | T: 0.171 | | | | |
| | TT | 0.019 | | | | | |
| | CC | 0.411 | C: 0.665 | 1.805 | 0.446 | 0.459 | 2.908 |
| scaffold17-796939 | CT | 0.506 | T: 0.335 | | | | |
| | TT | 0.082 | | | | | |
| | CC | 0.918 | C: 0.924 | 1.163 | 0.140 | 0.136 | 130.787 |
| scaffold18-2467783 | CT | 0.013 | T: 0.076 | | | | |
| | TT | 0.070 | | | | | |
| | CC | 0.044 | C: 0.237 | 1.567 | 0.362 | 0.878 | 0.698 |
| scaffold184-113778 | CT | 0.386 | T: 0.763 | | | | |
| | TT | 0.570 | | | | | |

| | | | | | | | |
|---|---|---|---|---|---|---|---|
| | AA | 0.006 | A: 0.051 | 1.106 | 0.096 | 0.993 | 0.970 |
| scaffold184-115262 | AG | 0.089 | G: 0.949 | | | | |
| | GG | 0.905 | | | | | |
| | CC | 0.810 | C: 0.905 | 1.221 | 0.181 | 0.166 | 1.738 |
| scaffold19-709400 | CT | 0.190 | T: 0.095 | | | | |
| | GG | 0.766 | G: 0.880 | 1.268 | 0.212 | 0.204 | 0.933 |
| scaffold2-2815335 | TG | 0.228 | T: 0.120 | | | | |
| | TT | 0.006 | | | | | |
| | CC | 0.981 | C: 0.991 | 1.019 | 0.019 | 0.019 | 0.015 |
| scaffold2-3426830 | CT | 0.019 | T: 0.009 | | | | |
| | AA | 0.013 | A: 0.120 | 1.268 | 0.212 | 0.963 | 0.046 |
| scaffold2-13687576 | AT | 0.215 | T: 0.880 | | | | |
| | TT | 0.772 | | | | | |
| | AA | 0.772 | A: 0.883 | 1.261 | 0.207 | 0.199 | 0.805 |
| scaffold20-453978 | AG | 0.222 | G: 0.117 | | | | |
| | GG | 0.006 | | | | | |
| | AA | 0.171 | A: 0.443 | 1.974 | 0.494 | 0.682 | 1.674 |
| scaffold21-4955509 | AG | 0.544 | G: 0.557 | | | | |
| | GG | 0.285 | | | | | |
| | AA | 0.006 | A: 0.136 | 1.307 | 0.235 | 0.954 | 1.698 |
| scaffold210-68511 | AG | 0.259 | G: 0.864 | | | | |
| | GG | 0.734 | | | | | |
| | AA | 0.209 | A: 0.335 | 1.805 | 0.446 | 0.788 | 3.472 |
| scaffold25-4206172 | AG | 0.380 | G: 0.665 | | | | |
| | GG | 0.475 | | | | | |
| | CC | 0.120 | C: 0.361 | 1.856 | 0.461 | 0.763 | 0.291 |
| scaffold290-11890 | CT | 0.481 | T: 0.639 | | | | |
| | TT | 0.399 | | | | | |
| | CC | 0.576 | C: 0.772 | 1.543 | 0.352 | 0.342 | 2.097 |
| scaffold3-16652593 | CT | 0.392 | T: 0.228 | | | | |
| | TT | 0.032 | | | | | |
| scaffold3-16653046 | CT | 0.228 | C: 0.114 | 1.253 | 0.202 | 0.194 | 2.612 |
| | TT | 0.772 | T: 0.886 | | | | |
| scaffold305-32581 | AG | 0.044 | A: 0.022 | 1.045 | 0.043 | 0.043 | 0.081 |
| | GG | 0.956 | G: 0.978 | | | | |
| | CC | 0.633 | C: 0.801 | 1.469 | 0.319 | 0.308 | 0.407 |
| scaffold31-9275712 | CT | 0.335 | T: 0.199 | | | | |
| | TT | 0.032 | | | | | |
| | AA | 0.006 | A: 0.054 | 1.128 | 0.114 | 0.992 | 0.719 |
| scaffold31-11182212 | AG | 0.095 | G: 0.940 | | | | |
| | GG | 0.892 | | | | | |
| | AA | 0.032 | A: 0.114 | 1.253 | 0.202 | 0.967 | 5.403 |
| scaffold31-12525471 | AC | 0.165 | C: 0.886 | | | | |
| | CC | 0.804 | | | | | |
| | AA | 0.051 | A: 0.225 | 1.535 | 0.348 | 0.889 | 0.0001 |
| scaffold34-3226435 | AC | 0.348 | C: 0.775 | | | | |
| | CC | 0.601 | | | | | |
| scaffold35-2164468 | AA | 0.114 | A: 0.367 | 1.868 | 0.465 | 0.757 | 1.270 |

| | | | | | | | |
|---|---|---|---|---|---|---|---|
| | AG | 0.506 | G: 0.633 | | | | |
| | GG | 0.380 | | | | | |
| | CC | 0.335 | C: 0.585 | 1.943 | 0.485 | 0.539 | 0.143 |
| scaffold36-4436178 | CT | 0.500 | T: 0.415 | | | | |
| | TT | 0.165 | | | | | |
| | AA | 0.291 | A: 0.516 | 1.998 | 0.499 | 0.609 | 1.592 |
| scaffold4-10430350 | AG | 0.449 | G: 0.484 | | | | |
| | GG | 0.259 | | | | | |
| | AA | 0.044 | A: 0.234 | 1.559 | 0.359 | 0.881 | 0.545 |
| scaffold435-12868 | AG | 0.380 | G: 0.766 | | | | |
| | GG | 0.576 | | | | | |
| | AA | 0.753 | A: 0.829 | 1.395 | 0.283 | 0.272 | 34.010 |
| scaffold49-4261152 | AG | 0.152 | G: 0.171 | | | | |
| | GG | 0.095 | | | | | |
| | CC | 0.797 | C: 0.899 | 1.223 | 0.182 | 0.176 | 2.006 |
| scaffold5-12042944 | CT | 0.203 | T: 0.101 | | | | |
| | AA | 0.006 | A: 0.111 | 1.245 | 0.197 | 0.968 | 0.574 |
| scaffold59-960994 | AG | 0.209 | G: 0.889 | | | | |
| | GG | 0.785 | | | | | |
| | AA | 0.108 | A: 0.304 | 1.733 | 0.423 | 0.818 | 0.827 |
| scaffold6-5210078 | AC | 0.392 | C: 0.696 | | | | |
| | CC | 0.500 | | | | | |
| | CC | 0.468 | C: 0.649 | 1.837 | 0.456 | 0.475 | 6.865 |
| scaffold6-9258715 | CT | 0.361 | T: 0.351 | | | | |
| | TT | 0.171 | | | | | |
| | CC | 0.108 | C: 0.253 | 1.608 | 0.378 | 0.864 | 8.364 |
| scaffold6-15802194 | CT | 0.291 | T: 0.747 | | | | |
| | TT | 0.601 | | | | | |
| | AA | 0.019 | A: 0.206 | 1.485 | 0.327 | 0.904 | 3.220 |
| scaffold65-275780 | AG | 0.373 | G: 0.794 | | | | |
| | GG | 0.608 | | | | | |
| | AA | 0.019 | A: 0.149 | 1.339 | 0.253 | 0.946 | 0.097 |
| scaffold66-172323 | AG | 0.259 | G: 0.851 | | | | |
| | GG | 0.722 | | | | | |
| | CC | 0.203 | C: 0.405 | 1.930 | 0.482 | 0.720 | 4.023 |
| scaffold69-513313 | CT | 0.405 | T: 0.595 | | | | |
| | TT | 0.392 | | | | | |
| | AA | 0.070 | A: 0.225 | 1.558 | 0.358 | 0.890 | 1.857 |
| scaffold7-15455173 | AG | 0.310 | G: 0.769 | | | | |
| | GG | 0.614 | | | | | |
| | CC | 0.152 | C: 0.430 | 1.962 | 0.490 | 0.695 | 2.920 |
| scaffold7-15455275 | CT | 0.557 | T: 0.570 | | | | |
| | TT | 0.291 | | | | | |
| | CC | 0.468 | C: 0.690 | 1.748 | 0.428 | 0.433 | 0.198 |
| scaffold7-20336421 | CT | 0.443 | T: 0.310 | | | | |
| | TT | 0.089 | | | | | |
| | CC | 0.614 | C: 0.753 | 1.592 | 0.372 | 0.364 | 9.956 |
| scaffold8-9389475 | CT | 0.278 | T: 0.247 | | | | |
| | TT | 0.108 | | | | | |
| scaffold8-12482471 | CC | 0.886 | C: 0.940 | 1.127 | 0.113 | 0.110 | 0.364 |
| | CT | 0.108 | T: 0.060 | | | | |

| | | | | | | |
|---|---|---|---|---|---|---|
| | TT | 0.006 | | | | |
| | CC | 0.557 | C: 0.753 | 1.592 | 0.372 | 0.364 | 0.485 |
| scaffold9-8466307 | CT | 0.392 | T: 0.247 | | | | |
| | TT | 0.051 | | | | | |
| | AA | 0.468 | A: 0.677 | 1.777 | 0.437 | 0.446 | 0.313 |
| scaffold9-8467739 | AG | 0.418 | G: 0.323 | | | | |
| | GG | 0.114 | | | | | |
| | AA | 0.127 | A: 0.174 | 1.404 | 0.288 | 0.928 | 70.884 |
| scaffold91-1266067 | AG | 0.095 | G: 0.826 | | | | |
| | GG | 0.778 | | | | | |

Note: $\chi 2$ value corresponds to *P* value *P*0.05(2) = 5.99, *P*0.01(2) = 9.21.
